# Supplementary material for: Enhanced Li-Ion Rate Capability and Stable Efficiency Enabled by MoSe2 Nanosheets in Polymer-Derived Silicon Oxycarbide Fiber Electrodes
Source: Nanomaterials (Basel). 2022 Feb 6;12(3):553. doi: 10.3390/nano12030553 (PMC8839961; doi:10.3390/nano12030553)
Supplement: Supplementary file 1 [file nanomaterials-12-00553-s001.zip › nanomaterials-1553535-SI.pdf]

Supplementary Materials

Enhanced Li-ion Rate Capability and Stable Efficiency Enabled by $MoSe_2$ Nanosheets in Polymer-derived Silicon Oxycarbide Fiber Electrodes

Sonjoy Dey *, Shakir Bin Mujib * and Gurpreet Singh

Department of Mechanical and Nuclear Engineering, Kansas State University, Manhattan, KS 66506, USA; gurpreet@ksu.edu

* Correspondence: sonjoy@ksu.edu (S.D.); sbmujib@ksu.edu (S.B.M.); Tel.: +1-785-770-5891 (S.D.); +1-785-410-5023 (S.B.M.)

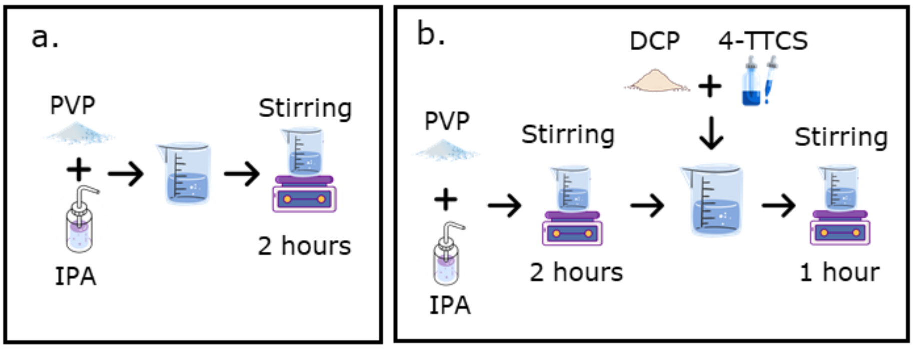

Figure S1: Solution preparation process of (a) PVP fibermat; (b) PVP/SiOC fibermat.

Table S1: Comparison of the electrochemical performance of the PVP/SiOC/$MoSe_2$ anode with previously studied SiOC anodes for LIBs.

Materials	Current Density (mA g^{-1})	Capacity (mAh g^{-1})	References
SiOC/$MoSe_2$	50, 100	586,393	This work
SiOC	37	540	[70]
SiOC	100	100	[71]
N-doped RGO aerogels with SiOC	148	565	[72]
SiOC/Graphene	50	582	[73]
SiOC	74	460	[74]
SiOC	36	150	[75]
SiOC	186	303	[58]
SiOC etched with HF	18	457	[60]
SiOC	50	578	[60]
SiOC	50	243	[60]
SiOC	50	53	[60]
SiOC	18.6	568	[76]
SiOC/Glucose	37	201	[13]
SiOC/Potato Starch	37	140	[13]
SiOC/Potato Starch	37	44	[13]
